# Editorial: Telestroke and stroke care networks beyond the comprehensive stroke center

**DOI:** 10.3389/fstro.2024.1501958

**Published:** 2024-10-24

**Authors:** Amanda Jagolino-Cole, Alicia Zha, Imama A. Naqvi, Jennifer J. Majersik

**Affiliations:** ^1^Department of Neurology, University of Texas Health Science Center at Houston, Houston, TX, United States; ^2^Department of Neurology, The Ohio State University, Columbus, OH, United States; ^3^Department of Neurology, Columbia University, New York, NY, United States; ^4^Department of Neurology, The University of Utah, Salt Lake City, UT, United States

**Keywords:** stroke, ischemic stroke, emergency medical services (EMS), telestroke, telemedicine, stroke centers, artificial intelligence, virtual reality

Telestroke is a tool that addresses structural and geographic equity in stroke care, globally. In the US, certified Comprehensive Stroke Centers make up only about five percent of acute stroke services (Boggs et al., 2022). Low-income countries have ~70% fewer stroke units, about a third fewer acute stroke treatments, and less rehabilitation and education services (Owolabi et al., 2021). Foundations in acute stroke management via traditional telestroke in the emergency setting has led to expansion of telestroke in other essential areas of stroke care including pre-hospital, inter-facility, rehabilitation, and ambulatory settings. However, consistent access to technological requirements across the stroke care continuum remains a challenge (Guzik et al., 2021). Optimizing telestroke utilization beyond the Comprehensive Stroke Center, and within a telestroke network, remains a vital mission. This collection of five articles fills in some of the gaps in this knowledge base.

## Pre-hospital telestroke

Prehospital triage of acute stroke patients involves complex medical decision-making and knowledge of regional hospital systems. In their reviews, Ranta et al. and Chapman et al. discuss the feasibility and rationale for use of telestroke in ambulance-based medicine. The remote presence of a neurologist, with specialized skills in comprehensive neurological examination and acute stroke management, can aid paramedics in facilitating timely diagnosis and triage, bypass, and treatment decisions. These pre-hospital steps can improve timing of critical stroke care steps in the Emergency Department including time to imaging, to intravenous reperfusion therapy, and to skin-puncture times for thrombectomy candidates.

Prehospital telestroke implementation requires specific considerations. Chapman et al. describe findings from pre-hospital telestroke studies performed in a variety of settings: simulated vs. actual patient scenarios, urban vs. suburban vs. rural areas, interfacility telestroke, and with or without mobile stroke units. They include clinician perspectives regarding satisfaction, acceptability of connectivity and technology, and usability in simulated encounters. Pre-hospital telestroke utilizes a variety of personnel as telepresenters, including paramedics, neurologists, and advanced practitioners, underscoring the importance of interprofessional training and collaboration in this setting.

Wider implementation of pre-hospital telestroke may reveal a new set of obstacles and mitigation strategies for which physicians will need to train. Adding pre-hospital telestroke to existing acute stroke care workflow requires strategy, as Ranta et al. indicate. Mobile stroke units may have a role in less densely populated areas such as rural populations but the impact is still unclear (Mathur et al., 2019). Regional and national governance may streamline workflow and algorithms. Chapman et al. points out how essential buy-in from local telecommunication service providers is, and that changes are needed at the policy level, especially for underserved populations. Both reviews conclude that determining cost-effectiveness, developing algorithms for triage, and strategizing to address local needs are essential for equitable implementation.

## System-level telestoke

Successful telestroke implementation relies on its ability to respond to changes in healthcare. Ryan et al. illustrate such evolution in their summary of responses to a continental telestroke. While overall there has been a linear growth in telestroke usage in Europe, telestroke networks established in prior to 2013 had more spoke sites than those established afterwards, experiencing a higher volume of consultations yearly. Perhaps larger hospitals had more ability to disseminate care via telestroke, so were earlier adopters. One could infer that smaller networks are more feasible now with new and improved technologies, and telestroke has led to decentralization of acute care services. Either way, more hospitals are being reached than previously with telestroke. The authors explain that while the overall primary use of telestroke was for diagnosis and triage, at least one network utilized telestroke for transient ischemic attack ambulatory services, demonstrating that it can differentially fill in stroke care gaps.

An Australian statewide telestroke network deployed in 2013 to serve rural hospitals demonstrated improved regional stroke metrics including door-to-decision and door-to-needle times, and proportion of patients receiving. Hospitals in this network were not Comprehensive Stroke Centers, so the proportion of patients transferred for thrombectomy was unchanged. The authors emphasized that while telestroke brings clinical stroke expertise to patients, patient care still ultimately relies on local providers and resources. As part of establishing their network, Garcia-Esperon et al. described the role of an effective educational intervention for spoke clinicians, novel for its use of virtual reality (VR). The ability to train and educate staff effectively in low-resource environments can be difficult, given the size and bandwidth of local workforces and geographic distances between hospitals, and robuse local education is essential. While the authors experienced challenges–including initiation delays, limited local uptake, and lack of longitudinal sustainability, they demonstrated feasibility. These VR sessions, which address patient assessment, communication workflows, imaging review, treatment decision-making, and patient education, were interactive, offered a more immersive and active learning approach.

Finally, Devlin et al. relay the role of artificial intelligence in supporting telestroke clinicians, using a care coordination and communication. They found implementation improved large vessel occlusion identification and neurointernventionalist notification in both thrombectomy capable and non-thrombectomy capable hospitals. Differences in baseline characteristics between sites that utilized the application and those that did not, were not explored. The presence of telestroke providers experienced in use of these technologies will be instrumental for broad uptake of their application.

Ultimately, across multiple settings, telestroke addresses inequalities in access to vascular neurologists. Telestroke mitigates delays in time-sensitive diagnosis and treatment for acute stroke patients, and optimize triage patterns and better allocate resources within and among stroke systems of care. We have yet to discover how best to utilize telehealth and telestroke within the continuum of care from primordial to secondary prevention, pre-hospital to ED settings, interhospital- and system-levels, and in ambulatory stroke, rehabilitation, and recovery. Randomized trials and guidance on metrics specific to settings outside of the ED, including pre-hospital and interhospital scenarios, are warranted. Patent-reported outcomes require further exploration. Commonly identified themes among the pool of research to date in telestroke are governmental buy-in and policy changes, community-specific algorithms, and more uniform connectivity and technology availability. Such data is still forthcoming in other parts of the world. Still, we hope our current collection of articles provides some insight into the potential of telestroke to streamline the stroke care continuum across globally underserved settings.
